# The Elegance of the Acyclic Nucleoside Phosphonates (ANPs), Honorary Tribute to Antonín Holý, Who Passed Away on 16 July 2012, at the 10th Anniversary of His Death

**DOI:** 10.3390/v14091978

**Published:** 2022-09-07

**Authors:** Erik De Clercq

**Affiliations:** Rega Institute for Medical Research, Herestraat 49, B-3000 Leuven, Belgium; erik.declercq@kuleuven.be

**Keywords:** HIV, ANPs, cidofovir, adefovir, tenofovir, TDF, TAF, HBV, PrEP

## Abstract

My collaboration with Prof. Antonín Holý, that spans a period of 3–4 decades (1976–2012), led to the discovery of several acyclic nucleoside phosphonates (ANPs) which were clinically developed by Gilead Sciences: cidofovir, adefovir, and tenofovir. The latter was further converted to two orally bioavailable prodrug forms, TDF and TAF, and both TDF and TAF were further combined with other antiviral drugs, thus giving rise to a broad array of antiviral drug combinations for the treatment of HIV infections. TDF and TAF are both available for the treatment of hepatitis B virus (HBV) infections, and, in combination with emtricitabine, also applicable as Truvada^®^ and Descovy^®^, respectively, for the prophylaxis of HIV infections.

## 1. Prelude: DHPA

**Figure 1:**
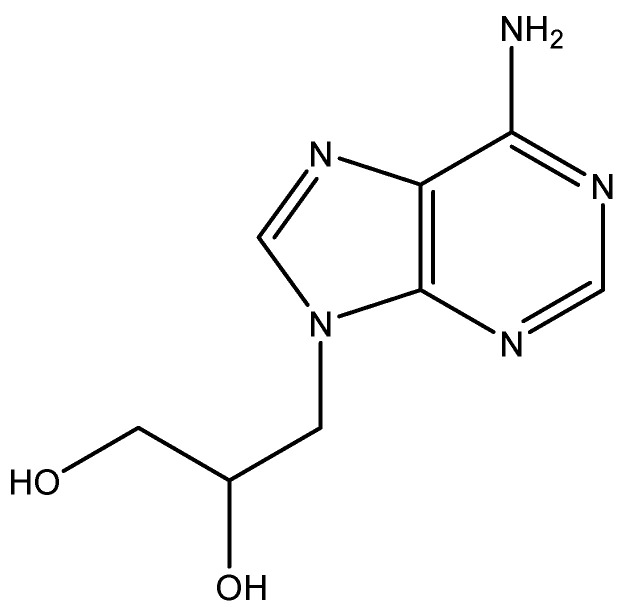
DHPA.

The first time I ever encountered Antonín Holý was on 3–5 May 1976, when we both attended the symposium on Synthetic Nucleosides, Nucleotides and Polynucleotides, 3–5 May 1976, organized by (the late) Karl-Heinz Scheit at the Max Planck Institut für Biophysikalische Chemie in Göttingen. Holý and I then agreed to start a collaboration of the antiviral activity of nucleoside analogues, and of the three compounds that were initially evaluated for activity, one proved effective. It was (*S*)-9-(2,3-dihydroxypropyl)adenine (Figure 1), dubbed DHPA, and published in *Science* [1]. DHPA was discovered shortly after acyclovir had been revealed as the first acyclic nucleoside analogue that was found to be specifically effective against herpesvirus, i.e., herpes simplex virus (HSV) [2], based on a specific recognition (i.e., phosphorylation) by the HSV-encoded thymidine kinase (TK) [3]. In contrast with acyclovir, which was specifically active against HSV type 1 (HSV-1) and type 2 (HSV-2) and other herpesviruses encoding a specific TK, DHPA had a broad-spectrum antiviral activity against both DNA viruses (i.e., vaccinia virus) and some RNA viruses (i.e., vesicular stomatitis virus (VSV)). Its action target would be recognized later as the S-adenosylhomocysteine (SAH) hydrolase, and in this capacity it interfered with the S-adenosylmethionine (SAM)-dependent methylation reaction such as those involved in the maturation of viral Mrna [4]. In addition to DHPA, several other acyclic and carbocyclic adenosine analogues have been identified as SAH hydrolase inhibitors and their antiviral potential has been extensively reviewed in a tribute to John A. Montgomery [5]. Whereas acyclovir (later succeeded by its oral prodrug, valacyclovir) became worldwide the gold standard for the treatment of HSV infections, DHPA was only temporarily commercialized in the Czechoslovak Republic (Ceskoslovensko) for topical treatment, as an ointment (Duviragel^®^) of herpes labialis (fever blisters, cold sores). Starting from DHPA, Holý synthesized several other aliphatic adenosine analogues which were all evaluated for their antiviral activity [6], but not commercialized for clinical use.

## 2. The First Acyclic Nucleoside Phosphonate (ANP): HPMPA

**Figure 2:**
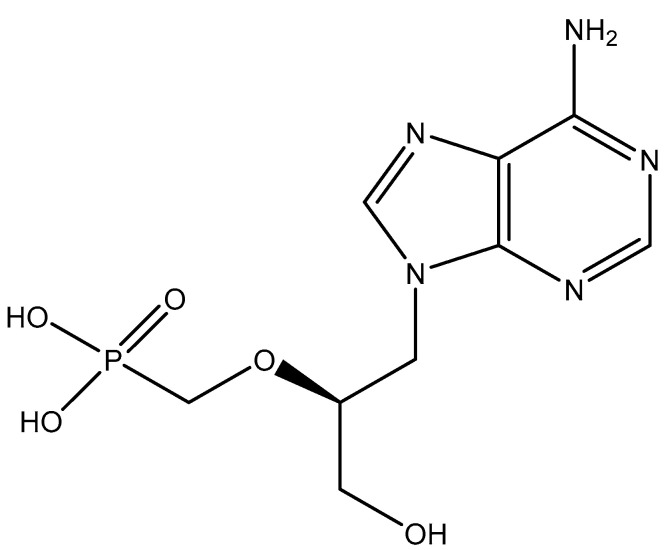
HPMPA.

HPMPA [(*S*)-9-(3-hydroxypropyl-2-methoxy-phosphonyl)adenine] was the first ANP analogue (Figure 2) ever shown to exhibit broad-spectrum activity against DNA viruses [7]. Its spectrum of activity included herpes-, adeno-, pox-, polyoma-, and papillomaviruses, and was later extended to the hepadnaviruses, i.e., hepatitis B virus (HBV) [8,9,10,11,12]. HPMPA could be viewed as a hybrid molecule between DHPA and phosphonoformic acid (PFA). The latter had earlier been described by B. Öberg [13] for its antiviral activity against a broad range of DNA viruses (to be later extended to retroviruses, i.e., HIV) and was commercialized (for intravenous infusion as Foscavir^®^) for the treatment of HSV infections resistant against acyclovir [14,15].

On a compassionate basis, HPMPA has been used successfully in the topical treatment of adenovirus conjunctivitis in humans, but like IDU (idoxuridine) and TFT (trifluridine), which are widely used topically for the local treatment of HSV keratoconjunctivitis in patients, HPMPA has not been marketed for systemic administration in any medical virus infection.

## 3. HPMPC, Cidofovir, Vistide^®^

**Figure 3:**
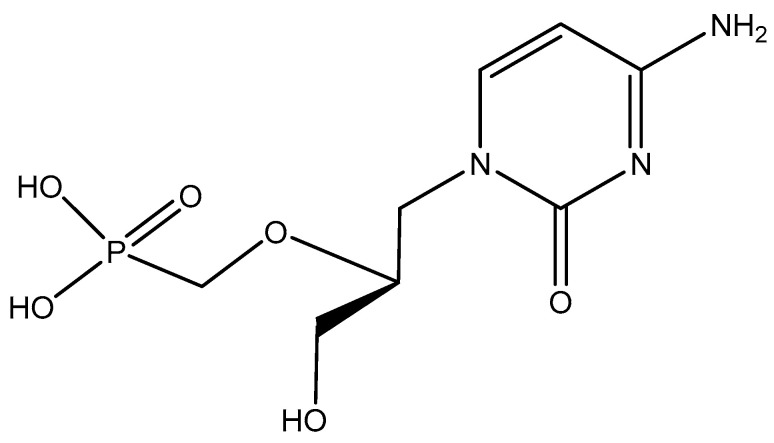
HPMPC (cidofovir, Vistide^®^).

Shortly after HPMPA, we described its cytosine counterpart, HPMPC [(*S*)-1-(3-hydroxypropyl-2-methoxyphosphonyl)cytosine] (Figure 3) for its broad-spectrum anti-DNA viral activity, similar to that of HPMPA [16]. In 1988, Snoeck et al. [17] further documented the potential of HPMPC (cidofovir) in the treatment of human cytomegalovirus (HCMV) infection, and, at Gilead Sciences, they further explored, with the help of several clinicians (including J.P. Lalezari), the clinical potential of cidofovir for the treatment of HCMV retinitis. In 1996, cidofovir (Vistide^®^) was approved by the US FDA (Food and Drug Administration) for clinical use in the (systemic) treatment of HCMV retinitis in AIDS patients. On a compassionate basis, cidofovir has also been successfully used in the treatment of various DNA virus infections other than HCMV, but it has not been formally approved for these indications.

Noteworthy is the potential usefulness of cidofovir in the treatment of monkeypox virus infections in humans. That cidofovir would be more efficacious than the smallpox vaccine for this indication has been experimentally ascertained in a previous study of Stittelaar et al. [18].

Cidofovir (Vistide^®^) is applicable by the intravenous route. To make it orally bioavailable, a prodrug (brincidofovir, hexadecyloxypropyl-cidofovir, CMX001) has been developed, and this compound should be amenable for the treatment of all DNA virus infections for which cidofovir itself is indicated.

## 4. Adefovir Dipivoxil (Hepsera^®^)

**Figure 4:**
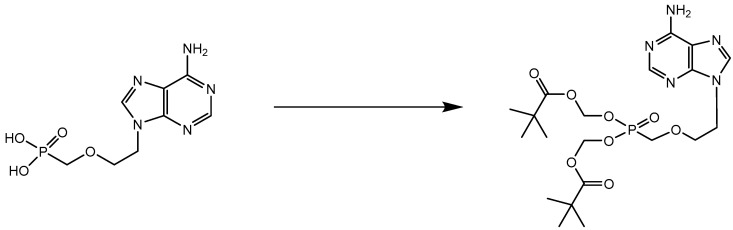
PMEA (adefovir) **→** Adefovir dipivoxil (Hepsera^®^).

**Figure 5:**
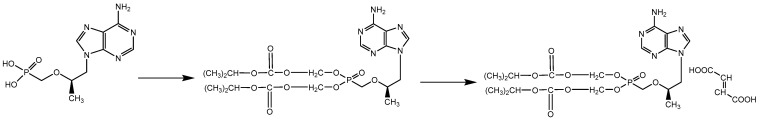
(*R*)-PMPA (Tenofovir) **→** Tenofovir disoproxil **→** Tenofovir disoproxil fumarate (TDF, Viread^®^).

In article [7] revealing HPMPA, a simpler, non-racemic ANP, PMEA [9-(2-phosphonylmethoxyethyl)adenine] was mentioned as an antiretroviral agent. This compound was further described by Pauwels et al. [19] and its mechanism of anti-HIV action resolved [20]. It was extensively evaluated for its potential in the treatment of HBV infections [10]. Its oral prodrug, adefovir dipivoxil (Figure 4), was first pursued for the treatment of HIV infections, but two observations discouraged this planning: first, the advent of tenofovir that, in its prodrug form, TDF (Figure 5), was more potent than adefovir dipivoxil; and second, adefovir dipivoxil was more effective against HBV at a lower dosage that that required for HIV treatment.

## 5. Tenofovir Disoproxil Fumarate (TDF)

Tenofovir, originally named (*R*)-PMPA, was first described in 1993 [21]. In terms of antiretroviral potency, it was even superseded by its 2,6-diaminopurine (DAP) analogue, but the latter was not chosen (by Gilead) for further development. Of crucial importance was the observation of Tsai et al. [22] showing that (*R*)-PMPA (tenofovir) was 100% effective in suppressing simian immunodeficiency virus (SIV) infection in rhesus macaques if treatment was given shortly (within a few hours or days) before or after SIV infection. Further studies by Robbins et al. [23] and Naesens et al. [24] identified the disoproxil derivative of tenofovir as its orally bioavailable prodrug which was then further converted to its fumarate to be approved and marketed as tenofovir disoproxil fumarate (TDF, Viread^®^) (Figure 5). 

## 6. Drug Combinations Containing TDF

TDF was approved in 2001 by the US FDA for the treatment of HIV infections. Then followed in 2004 the approval of the combination of TDF and emtricitabine [(-)FTC] (Truvada^®^), in 2006 the combination of TDF with emtricitabine and efavirenz (Atripla^®^), in 2011 the combination of TDF with emtricitabine and rilpivirine (Complera^®^ (US) and Eviplera^®^ (EU)), and in 2012 the combination of TDF with emtricitabine, elvitegravir, and cobicistat (Stribild^®^). That drug combination therapy was advocated for the therapy of HIV infections stemmed from the long-term drug combination strategy installed in the therapy of Mycobacterium tuberculosis infections. This strategy is aimed at three principles: (i) to obtain synergism between different compounds interacting with different sites of the life cycle of the microorganism, (ii) to lower the individual drug doses and the herewith associated toxic side effects, and (iii) to diminish the likelihood of drug resistance development. This drug combination strategy has been widely implemented for the antiviral drug therapy of HIV infections [25].

## 7. Tenofovir Alafenamide (TAF)

**Figure 6:**
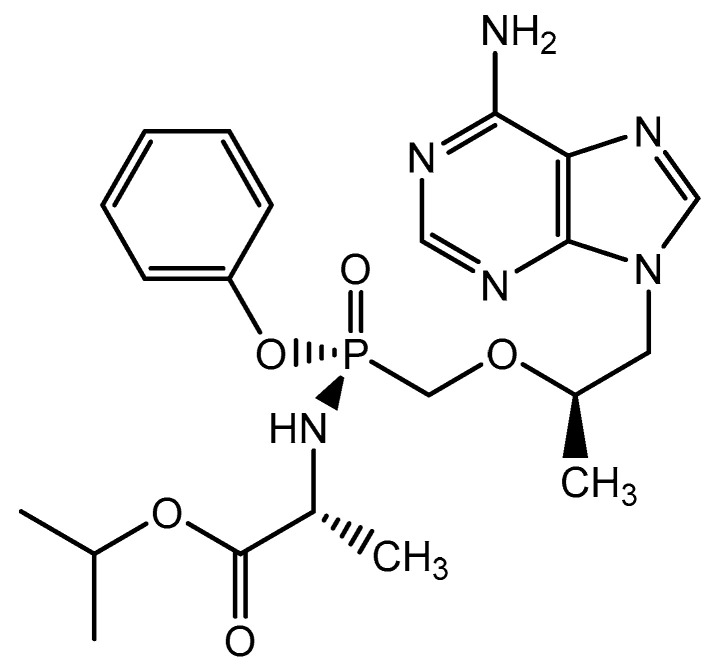
Tenofovir alafenamide (TAF, Vemlidy^®^).

Following tenofovir disoproxil fumarate (TDF), another orally bioavailable prodrug of tenofovir, namely tenofovir alafenamide (TAF, GS-7340) (Figure 6) was developed for the treatment of HIV infections [26]. As compared to TDF, TAF is preferentially taken up by the lymphoid cells, the principal target cells for the replication of HIV. This allows a *circa* 10-fold reduction in the daily dosage of tenofovir, thereby reducing the liabilities inherently linked to the administration of tenofovir, i.e., nephrotoxicity and bone demineralization. Thus, TAF is endowed with a better safety profile than TDF as far as the kidney functions and bone stability are concerned.

## 8. Drug Combinations Containing TAF

Akin to TDF, TAF has also been formulated with several other antiviral drugs, and these drug combinations were successively approved by the US FDA in 2015 (Genvoya^®^: TAF, emtricitabine, elvitegravir and cobicistat), in 2016 (Odefsey^®^: TAF, emtricitabine and rilpivirine), in 2018 (Biktarvy^®^: TAF, emtricitabine and bictegravir) and still in 2018 (Symtuza^®^: TAF, emtricitabine, darunavir and cobicistat).

## 9. Treatment of HBV (Hepatitis B Virus) Infections

While PMEA (adefovir) in its oral prodrug form adefovir dipivoxil (Hepsera^®^) was the first ANP ever to be licensed for clinical use in the treatment of HBV infections, two other ANP prodrugs have in the meantime been added to the anti-HBV drug armamentarium: TDF (Viread^®^) and TAF (Vemlidy^®^). To which extent Viread^®^ and Vemlidy^®^ require prolonged, if not life-long treatment of chronic hepatitis B and/or could eventually achieve complete sterilization (i.e., elimination of HBV) needs to be further evaluated.

## 10. Prophylaxis of HIV Infections [Pre-Exposure Prophylaxis (PrEP)]

That an effective vaccine against HIV infection could ever be developed has become increasingly enigmatic, if not utopian. Meanwhile, chemoprophylaxis has gained alternative acceptance, and two drug combinations (i.e., Truvada^®^ (TDF plus emtricitabine) and Descovy^®^ (TAF plus emtricitabine)) are currently available for this purpose. These drug combinations can be administered orally once daily to prevent HIV infection from occurring (PrEP). The US FDA approved the prophylactic use of Truvada^®^ on 16 July 2012, exactly the same day that Antonín (“Tonda”) Holý died. In recognition of what he has accomplished at the service of human health, I feel honored and grateful to dedicate this article as a tribute to Tonda, my dearest friend, to commemorate the tenth anniversary of his passing away.

## Data Availability

Not applicable.
